# A three-stage approach for genome-wide association studies with family data for quantitative traits

**DOI:** 10.1186/1471-2156-11-40

**Published:** 2010-05-14

**Authors:** Ming-Huei Chen, Martin G Larson, Yi-Hsiang Hsu, Gina M Peloso, Chao-Yu Guo, Caroline S Fox, Larry D Atwood, Qiong Yang

**Affiliations:** 1 Department of Neurology and Framingham Heart Study, Boston University School of Medicine, Boston, MA, USA; 2 The NHLBI's Framingham Heart Study, Framingham, MA, USA; 3 Department of Mathematics and Statistics, Boston University, Boston, MA, USA; 4 Genetic Epidemiology Program, Hebrew Senior Life Institute for Aging Research and Harvard Medical School, Boston, MA, USA; 5 Molecular and Integrative Physiological Sciences Program, Harvard School of Public Health, Boston, MA, USA; 6 Department of Biostatistics, Boston University School of Public Health, Boston, MA, USA; 7 Clinical Research Program, Children's Hospital Boston, Boston, MA, USA; 8 Program in Genomics, Department of Medicine, Children's Hospital Boston, Boston, MA, USA; 9 Department of Pediatrics, Harvard Medical School, Boston, MA, USA; 10 The Center for Population Studies, National Heart, Lung, and Blood Institute, Bethesda, MD, USA

## Abstract

**Background:**

Genome-wide association (GWA) studies that use population-based association approaches may identify spurious associations in the presence of population admixture. In this paper, we propose a novel three-stage approach that is computationally efficient and robust to population admixture and more powerful than the family-based association test (FBAT) for GWA studies with family data.

We propose a three-stage approach for GWA studies with family data. The first stage is to perform linear regression ignoring phenotypic correlations among family members. SNPs with a first stage p-value below a liberal cut-off (e.g. 0.1) are then analyzed in the second stage that employs a linear mixed effects (LME) model that accounts for within family correlations. Next, SNPs that reach genome-wide significance (e.g. 10^-6 ^for 34,625 genotyped SNPs in this paper) are analyzed in the third stage using FBAT, with correction of multiple testing only for SNPs that enter the third stage. Simulations are performed to evaluate type I error and power of the proposed method compared to LME adjusting for 10 principal components (PC) of the genotype data. We also apply the three-stage approach to the GWA analyses of uric acid in Framingham Heart Study's SNP Health Association Resource (SHARe) project.

**Results:**

Our simulations show that whether or not population admixture is present, the three-stage approach has no inflated type I error. In terms of power, using LME adjusting PC is only slightly more powerful than the three-stage approach. When applied to the GWA analyses of uric acid in the SHARe project of FHS, the three-stage approach successfully identified and confirmed three SNPs previously reported as genome-wide significant signals.

**Conclusions:**

For GWA analyses of quantitative traits with family data, our three-stage approach provides another appealing solution to population admixture, in addition to LME adjusting for genetic PC.

## Background

Many published genome-wide association (GWA) studies with dense SNP markers are based on study designs with unrelated individuals. It is well known that such designs are prone to spurious association caused by population admixture. A current popular solution to this problem involves identifying axes of genetic variation via principal components (PC) analyses with a large number of SNPs and then adjusting for the PC in the association analysis [1]. However, this approach may fail to adjust for population stratification in some cases [2]. Family-based designs, including Transmission Disequilibrium Test (TDT) and Family-based Association Test (FBAT) [3,4] are inherently robust to population admixture by conditioning on parental genotype data. As a result, parental data do not contribute to the power of the test, nor do the families with both non-informative (homozygous) parents. Therefore, FBAT has lower power in a homogeneous population than population-based association approaches that use all genotyped and phenotyped individuals.

In the context of GWA studies with family data, we present three basic strategies to handle a family-based design, and then we use the three basic strategies to form a two-stage population-based approach, and a three-stage family-based approach. The two-stage approach is computationally efficient and can account for familial relatedness, whereas the three-stage approach is additionally robust to population admixture. We performed simulations to evaluate the three basic strategies, the two-stage and the three-stage approaches. We also applied these strategies to Framingham Heart Study (FHS) SNP Health Association Resource (SHARe) 550K GWA analyses of uric acid.

## Methods

### Least squares regression

Least squares regression (denoted LM) is available in many standard statistical software packages and is fast to compute. The latter property is particularly desirable for the latest GWA studies with growing number of SNPs and phenotypes to analyze. The LM model can be expressed as

where *Y*_*ij *_and *G*_*ij *_are the phenotype and genotype for the *j*^*th *^person in the *i*^*th *^family, respectively. *X*(.) is the coding of the genotype and *ε*_*ij*_'s are independent random measurement errors following a Gaussian distribution. *β *can be estimated via maximum likelihood assuming that *Y*_*ij*_'s are independent and the p-value of testing genetic association is computed via two-sided t test. However, for family-based design, *Y*_*ij*_'s from the same family are correlated due to environmental and genetic effects. LM assumes independent *Y*_*ij*_'s, which could result in inflated type I error due to unexplained phenotype correlations in a family.

### Linear mixed effects model

Linear mixed effects (LME) model is traditionally used to model correlated continuous outcomes. LME is a generalization of LM with an additional random intercept that can account for familial correlation in *Y*, i.e.

where  are subject specific random effects correlated within a family,  is the variance due to these effects assuming homogeneous variance among different individuals, and Σ_*i *_is the correlation matrix in *i*^th ^family. Similar to [5], we assume that Σ_*i *_is the matrix of the coefficient of relationships or twice the coefficient of kinships, thus  are equivalent to the residual polygenic effects commonly modelled in familial aggregation or variance components linkage analyses [6], in which the residual correlation among family members are mostly due to small effects of many genes shared. The p-value of testing genetic association is computed via one-sided Wald chi-square test.

### Family-based association test

FBAT statistics [4] are based on the fact that, under the null hypothesis of no linkage and no association, conditional on parental genotypes, the transmission of parental alleles follows the Mendelian law of transmission. The test statistic takes the following form

where *P_i _*are the parental genotypes of the *i^th ^*family, *X*(*G*_*ij*_) is the score of the *j^th ^*child's genotype in the *i^th ^*family, and *E*(*X*(*G*_*ij*_)|*P*_*i*_) is the expected score of the child's genotype given parental genotype data. The p-value of testing genetic association is computed via two-sided Z test.

### Two-stage population-based approach

We propose a two-stage population-based approach for family data. The first stage is LM ignoring the familial correlations. The second stage uses LME on SNPs with first stage LM p-value less than α_1 _(for example, α_1 _= 0.1). This approach uses every genotyped and phenotyped individual and accounts for differential pedigree structures in the data. In addition, the first stage's screening makes this approach more efficient when applied to GWA studies. However, it is subject to the confounding due to population admixture.

### Three-stage family-based approach

**Figure 1:**
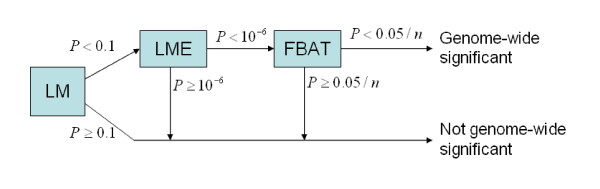
**Flow chart of the three-stage approach**. P-value cut-off is 0.1, 10^-6 ^and 0.05/*n *for the first, second and third stage, respectively, where *n *is the number of SNPs detected at the second stage LME. When FBAT is not included, the three-stage approach reduces to two-stage approach.

To control for population admixture, we further propose a three-stage family-based approach. The first two stages are identical to the two-stage approach proposed above. In the third stage, FBAT is conducted on SNPs with second stage p-value less than a specified threshold. FBAT p-value with multiple testing correction (such as Bonferroni correction) that only pertains to SNPs entered the third stage is used as final p-value for this approach. SNPs that did not enter the third stage are deemed as not associated with the phenotype of interest. This approach can be viewed as a special case of weighted FBAT approach [7], where SNPs that did not pass first two stages are assigned a weight of zero, and SNPs that entered third stage are assigned equal weights that summed up to one. Figure 1 presents a flow chart of the three-stage approach. The p-value cut-off at each stage is used in some of our simulations and *n *is the number of SNPs detected at the second stage LME. When FBAT is not included, the strategy reduces to two-stage approach.

### Simulation studies

We conducted simulation studies to evaluate the type I error and power of each of the introduced approaches. We simulated phenotype and/or genotype data, based on the real pedigree structures of 8,481 individuals from 1,494 pedigrees in the FHS SHARe project. Continuous traits are randomly generated following a multivariate normal distribution by using the program SOLAR [8], where the trait variance contains a quantitative trait locus (QTL), a polygenic and a residual variance component. The correlation between a pair of individuals in a pedigree due to the polygenic effect is twice their kinship coefficient. The QTL is assumed to be di-allelic, and the genotypes of founders are simulated under Hardy-Weinberg equilibrium (HWE). The offspring genotypes are simulated under the Mendelian law of transmission. The additive genetic model was used in both simulation and analysis.

For the basic strategies and the two-stage approach, combinations from the following simulation parameters are considered for evaluating type I error rates (Tables 1, 2 and 3): 1) minor allele frequency (MAF) 0.005, 0.01, 0.05, and 0.1; 2) polygenic heritability 0.3 and 0.6; 3) traits distributed as Normal, or non-Normal such as Chi-squared, absolute Normal, and Log-Normal.

**Table 1:** Type I error estimate at alpha = 0.05 with 10,000 replicates for quantitative phenotype and single SNP genotype data with MAF = 0.1.

Phenotype Distribution	Polygenic Variance	LM	LME	FBAT	LM-LME 1	LM-LME 2
Normal	0.3	0.084	0.051	0.050	0.049	0.051
Normal	0.6	0.115	0.052	0.049	0.046	0.050
Abs Normal	0.3	0.055	0.053	0.049	0.053	0.053
Abs Normal	0.6	0.064	0.050	0.049	0.050	0.050
Chi-square(1)	0.3	0.076	0.052	0.050	0.052	0.052
Chi-square(1)	0.6	0.104	0.049	0.051	0.045	0.047
Lognormal	0.3	0.059	0.054	0.046	0.054	0.054
Lognormal	0.6	0.066	0.049	0.047	0.049	0.049

The SNP explains 0% phenotype variation.

Normal: marginal phenotype follows the standard normal distribution

Abs Normal: marginal phenotype follows the absolute standard normal distribution

Chi-square(1): marginal phenotype follows a Chi-square distribution with 1 degree of freedom

Lognormal: marginal phenotype follows a Lognormal distribution

LM_LME1: two-stage approach with LM (p-value cut-off 0.1) at first stage

LM_LME2: two-stage approach with LM (p-value cut-off 0.2) at first stage

**Table 2:** Type I error estimate at alpha = 0.05 with 10,000 replicates of phenotype and a single SNP in LE with a QTL explaining 10% phenotype variation.

MAF	Polygenic Variance	LM	LME	FBAT	LM-LME 1	LM-LME 2
0.005	0.3	0.087	0.046	0.052	0.044	0.045
0.005	0.6	0.118	0.053	0.047	0.046	0.050
0.01	0.3	0.089	0.050	0.049	0.048	0.050
0.01	0.6	0.122	0.049	0.048	0.044	0.048
0.05	0.3	0.087	0.048	0.052	0.047	0.048
0.05	0.6	0.118	0.050	0.050	0.043	0.047
0.1	0.3	0.082	0.044	0.053	0.041	0.043
0.1	0.6	0.122	0.049	0.054	0.043	0.047

The SNP explains 0% phenotype variation.

A QTL is simulated to explain 10% phenotype variation and in LE with the SNP.

Marginal phenotype distribution follows the standard normal distribution.

LM_LME1: two-stage approach with LM (p-value cut-off 0.1) at first stage

LM_LME2: two-stage approach with LM (p-value cut-off 0.2) at first stage

**Table 3:** Correlation coefficient between FBAT and LME statistics based on 10,000 replicates of no SNP association with a continuous phenotype (marginal phenotype distribution follows the standard normal distribution).

		Correlation of FBAT and LME statistics at LME p-value level					
**MAF**	**Polygenic Variance**	**<.01**	**[.01-.05)**	**[.05-.1)**	**[.1-.2)**	**[.2-.3)**	**>.3**
0.01	0.3	0.89	0.82	0.77	0.68	0.61	0.35
0.01	0.6	0.91	0.83	0.79	0.69	0.63	0.36
0.05	0.3	0.87	0.81	0.75	0.71	0.61	0.34
0.05	0.6	0.89	0.84	0.76	0.72	0.64	0.36
0.1	0.3	0.90	0.81	0.77	0.70	0.60	0.34
0.1	0.6	0.90	0.83	0.79	0.73	0.63	0.36

The validity and power of the proposed three-stage approach can only be evaluated with genome-wide data, thus we conducted simulations with simulated phenotype and real SNP genotype data on chromosome 1 from the FHS's SHARe project. We simulated 100 datasets of phenotypes following normal distribution. Each phenotype dataset is combined with real genotype data of 34,265 SNPs on chromosome 1 that have passed quality control (HWE p-value > 1e-6, call rate>95%, MAF>0.01). We used simulated normal phenotypes with QTL variance 0, polygenic heritability 0.3 and 0.6, to estimate type I error rate for each studied method (Table 4). To assess power using real genotype data, we simulated the phenotype data by assuming SNP rs1570092 on chromosome 1 is the QTL that explains 1% of total phenotypic variance. The polygenic heritability is assumed to be 0.3. This SNP, rs1570092, was selected as the QTL on the bases of 1) HWE p-value 0.656; 2) call rate 100%; 3) MAF 0.31; and 4) in various levels of LD with some other SNPs on chromosome 1. The pair-wise LD measure (*r*^2 ^between any two SNPs on chromosome 1 was computed using R "genetics" package [9]. The SNPs were classified into five groups based on pair-wise *r*^2 ^between rs1570092 and other chromosome 1 SNPs for studying the power under different *r*^2^s (Table 5).

**Table 4:** Type I error estimate at alpha = 1e-6 using 100 replicates of phenotype without QTL effect and real 550K genotype data of 34,265 SNPs (HWE p-value > 1e-6, call rate > 95%, MAF > 0.01) on chromosome 1.

MAF	Polygenic Variance	LM	LME	FBAT	LM-LME 1	Three-stage
0.1	0.3	0.22	0.01	0.02	0.01	0.01

0.1	0.6	0.78	0.01	0.02	0.01	0

Marginal phenotype distribution follows the standard normal distribution.

LM_LME1: two-stage approach with LM at first stage with p-value cut-off 0.1 and LME at second stage with p-value cut-off 1e-6.

Three-stage: LM at first stage with p-value cut-off 0.1, LME at second stage with p-value cut-off 1e-6 and FBAT at third stage with p-value cut-off 0.05/*n*, where *n *is the number of SNPs entering the third stage.

**Table 5:** Power estimate at alpha = 1e-6 using 100 replicates of phenotype with 1% QTL (rs1570092 with MAF 0.31) effect and 30% of polygenic effect, and real 550K genotype data of 34,265 SNPs (HWE p-value > 1e-6, call rate > 95%, MAF > 0.01) on chromosome 1.

LD group	# SNPs in LD group	LM	LME	FBAT	LM-LME 1	Three-stage
0 <*r*^2 ^≤ 0.01	34025	0.20	0	0.01	0	0
0.01 <*r*^2 ^≤ 0.1	209	0.02	0.01	0	0.01	0
0.1 <*r*^2 ^≤ 0.3	6	0.70	0.54	0	0.54	0.50
0.3 <*r*^2 ^≤ 0.8	15	1	1	0.39	1	0.99
0.8 <*r*^2 ^≤ 1	10	1	1	0.51	1	0.99

rs1570092 explains 1% phenotype variation.

Marginal phenotype distribution follows a normal distribution with variance 1. The additive genetic effect is sqrt(0.01/2/0.31/0.69) = 0.153.

LM_LME1: two-stage approach with LM at first stage with p-value cut-off 0.1, and LME at second stage with p-value cut-off 1e-6.

Three-stage: LM at first stage with p-value cut-off 0.1, LME at second stage with p-value cut-off 1e-6 and FBAT at third stage with p-value cut-off 0.05/*n*, where *n *is the number of SNPs entering the third stage.

In addition, to evaluate the robustness and power of the three-stage approach in the presence of population admixture, we simulated phenotype and genotype of 100 admixture SNPs. We first partitioned the FHS SHARe population into two similar-size subpopulations with different allele frequencies and phenotypic means. For these 100 admixture SNPs, the allele frequencies in the two subpopulations were produced by Balding and Nichols approach [7,10] that uses a Beta distribution with mean *p *and variance *p*(1 - *p*)*F*_*ST*_. *F*_*ST*_, the "fixation index", was also a scaled variance of the subpopulation allele frequencies [11]. The combination of different allele frequencies and phenotypic means in subpopulations creates spurious associations in population-based association methods. In this simulation (100 replicates), the expected allele frequency *p *of the 100 admixture SNPs is 0.3; the QTL variance are 0 and 0.005 for assessing type I error and power, respectively; the polygenic variance is 0.3; *F*_*ST *_is 0.025; and the difference in phenotypic means is created by adding an offset value *δ *0.25 to phenotypic value in one subpopulation. Power is evaluated separately for detecting SNPs in LD (*r*^2 ^= 0.5, 0.8, 1) with the QTL and the QTL SNP. The software Eigenstrat [1] is used with 1) 34,625 SNPs and 100 simulated admixture SNPs, and 2) 100 simulated admixture SNPs, to obtain 10 PC that explain genetic ancestry. The original simulated phenotypes and residual phenotypes adjusting for PC obtained from 1) and 2) are analyzed. We are specifically interested in learning if the type I error rates of LME will be different in analyzing the two sets of residuals.

LME analyses were conducted using the lmekin function in the R GWAF package [12] modified from the same function in the R kinship package [13]. To test the fix effects, GWAF package uses the Wald Chi-square test, while kinship package uses t test. FBAT was computed using the FBAT program [4].

## Results

### Simulation studies

Table 1 presents the type I error rates of the three basic strategies and the two-stage approach at 0.05 significance level based on 10,000 replicates for a SNP with MAF 0.1, without a QTL affecting the phenotype. For the two-stage approach, we used two p-value cut-offs (0.1 and 0.2) at the first stage to evaluate the type I error rates. Except for LM, all methods have type I error rates close to 0.05 even when the phenotype does not follow a normal distribution for the scenarios we considered. For LM, the inflation increases with polygenic variance. For the two-stage approach, when the p-value cut-off at the first stage is more liberal, the type I error rate is slightly less conservative and closer to the type I error of LME. When considering a SNP with MAF of 0.01 and keeping other parameters constant, the results are similar to what we observed in Table 1 (results not shown). In brief, FBAT, LME and the two-stage approach are robust to MAF as low as 0.01 and to non-normality for the scenarios we considered, and only LM has inflated type I error rates.

Table 2 presents type I error rates of the three basic strategies and the two-stage approach at 0.05 significance level based on 10,000 replicates for SNPs with MAF 0.1, 0.05, 0.01 and 0.005, in linkage equilibrium (LE) with a QTL explaining 10% phenotype variation. LM has inflated type I error rates, while LME and FBAT have type I error rates close to 0.05. The two-stage approach seems to be slightly conservative, especially when using 0.1 p-value cut-off for the first stage. The results indicate that, even though there is unexplained large QTL variation in the phenotypes, LME still has correct type I error rates for SNPs with low MAF.

To study the relationship between FBAT and LME statistics under the null hypothesis of no association, we present in Table 3 the correlation coefficients between FBAT and LME statistics based on 10,000 replicates simulated similarly as in Table 1. We found FBAT and LME statistics are highly correlated under the null hypothesis, and the correlation is higher when LME p-value is smaller. For the considered non-Normal phenotypes, the results are similar, except that the correlations between the two statistics are weaker. Because of this, it may be impossible to obtain the type I error rate analytically using LME and FBAT sequentially to identify SNPs. When applied to GWA analyses, our proposed three-stage approach ensures valid global type I error by first controlling genome-wide type I error for the first two stages to 0.05 when there is no population admixture, and then controlling for population admixture by using FBAT at alpha = 0.05 via Bonferroni correction or other empirical p-value threshold for all SNPs reaching genome-wide significance in LME.

Table 4 presents type I error rates of basic strategies, two-stage and three-stage approaches estimated at 10^-6 ^genome-wide significance level (Bonferroni correction) using 100 replicates of Normal phenotypes without QTL (MAF 0.1) effect and real genotype data of 34,265 SNPs. The type I error rates are estimated by replicate. We use 0.01, 10^-6^, and 0.05/*n *as the p-value cut-offs for the first, the second and the third stage, respectively, where *n *is the number of SNPs entering the third stage. We choose 0.1 as the p-value cut-off at the first stage because it gives slightly more conservative type I error rate than 0.2 does, as shown in Tables 1 and 2. In addition, using smaller p-value cut-off at the first stage and keeping reasonable type I error can save computation time in GWA analyses. Only LM has inflated type I error rates, and all other approaches have slightly conservative type I error rates, which may be due to the conservative Bonferroni correction. Again, the inflation of LM's type I error increases with the unexplained familial correlation.

Table 5 presents the power and type I error rates using 100 replicates of Normal phenotypes with a QTL (rs1570092) effect of 1% and 34,265 SNPs, classified into five groups according to their *r*^2 ^with rs1570092. The power and type I error rates of each LD group are estimated at 10^-6 ^significance level by replicate. There is no inflated type I error (0 <*r*^2 ^≤ 0.01 group) for all compared methods except for LM. In general, the power increases as *r*^2 ^increases as expected, except some fluctuations are observed in LM and FBAT, which may be due to the large number of SNPs in the 0 <*r*^2 ^≤ 0.01 group. When *r*^2 ^> 0.3, LM, LME and the two-stage approach have 100% power to detect the association between the tested SNP and the simulated phenotype; while the three-stage approach and FBAT have about 99% and 39-51% power, respectively. The three-stage approach has shown to have similar power as LME and to be more powerful than FBAT.

Even though the three-stage approach is slightly less powerful than LME and the two-stage approach as shown in Table 5, it inherits the robustness to population admixture from FBAT. Therefore, we further consider population admixture (*F*_*ST *_= 0.025 and *δ *= 0.25) in our simulations where the type I error and power estimates are assessed at 10^-6 ^significance level by replicate (Table 6). Three phenotypes are analyzed in this simulation: "original phenotypes" = the original simulated Normal phenotypes; "residuals (1)" = residuals from adjusting the original simulated phenotypes for 10 Eigenstrat PC obtained from 34,625 SNPs and 100 admixture SNPs; and "residuals (2)" = residuals from adjusting the original simulated phenotypes for 10 Eigenstrat PC obtained from 100 admixture SNPs. Since FBAT and the three-stage approach are robust to the population admixture, we did not apply these two approaches to analyze residuals (1) and (2). Inflated type I error rates are observed in all studied population-based approaches (LM, LME and two-stage approach) when using the original phenotypes and even residuals (1). For FBAT and the three-stage approach, no inflated type I error rate is observed from analyzing the original simulated phenotypes. When using residuals (2), no inflation in type I error is observed for LME and the two-stage approach. When estimating power, 34,625 SNPs are not used. In general, LME and the two-stage approach using original simulated phenotypes and residuals (1) have similar power and are most powerful but with slightly inflated type I error, followed by the three-stage approach, LME and the two-stage approach with residuals (2), and then FBAT.

**Table 6:** Type I error and power estimates in the presence of population admixture at alpha = 1e-6 using 100 replicates.

	Phenotype data	LM	LME	FBAT	LM-LME 1	Three-stage
Type I error (r2 = 0)	original phenotypes	0.63	0.08	0.02	0.08	0.02
	
	residuals (1)	0.59	0.07	-	0.07	-
	
	residuals (2)	0.13	0.01	-	0.01	-

Power r2 = 0.5	original phenotypes	0.43	0.39	0	0.39	0.31
	
	residuals (1)	0.45	0.37	-	0.37	-
	
	residuals (2)	0.17	0.14	-	0.14	

Power r2 = 0.8	original phenotypes	0.66	0.63	0.05	0.63	0.56
	
	residuals (1)	0.66	0.63	-	0.63	-
	
	residuals (2)	0.57	0.47	-	0.47	-

Power QTL	original phenotypes	0.81	0.85	0.11	0.85	0.83
	
	residuals (1)	0.82	0.84	-	0.84	-
	
	residuals (2)	0.81	0.67	-	0.67	-

Marginal phenotype distribution follows a normal distribution with variance 1. The additive genetic effect in assessing power is sqrt(0.005/2/0.3/0.7) = 0.109.

LM_LME1: two-stage approach with LM at first stage with p-value cut-off 0.1 and LME at second stage with p-value cut-off 1e-6.

Three-stage: LM at first stage with p-value cut-off 0.1, LME at second stage with p-value cut-off 1e-6 and FBAT at third stage with p-value cut-off 0.05/*n*, where *n *is the number of SNPs entering the third stage.

The original simulated phenotypes and the residuals adjusted for 10 PC obtained from all 34,625 SNPs on chromosome 1 and 100 admixture SNPs (1), and from 100 admixture SNPs (2) are analyzed. The 100 admixture simulated SNPs have expected allele frequency 0.3, *F*_*ST *_= 0.025, the offset value *δ *= 0.25, QTL variance is 0.005, and the polygenic variation is 0.3. When estimating power, 34,265 SNPs are not used.

### Application to GWA analyses of uric acid level

We applied the strategies to the GWA analyses of uric acid levels in FHS SHARe project. A genome-wide scan of Affymetrix 550K SNP GeneChip on about 9,000 subjects was performed. GWA analyses using LME have identified 3 loci SCL2A9, ABCG2 and SCL17A3 with genome-wide significance (p-value < 5 × 10^-8^), and all the loci have been replicated in two other independent cohorts, the Rotterdam Study and the ARIC Study [14]. Here we re-analyze the residuals obtained from adjusting multi-variables for uric acid levels using the three-stage approach. The original FHS uric acid levels phenotype is approximately normal distributed with mean 315.2 and standard deviation 89.2 in μmol/l [14]. We first analyze all 550K SNPs using LM. With a p-value cut-off of 0.1, 80,527 SNPs passed the first stage and were analyzed by LME. Among them, 150 SNPs reached the genome-wide significance level. The 150 SNPs are located in the same three loci identified previously by LME [14]. When using 0.05/150 as significance level for the third stage FBAT analyses, 115 SNPs in SCL2A9 and ABCG2 reach the significance level. If only the most significant SNPs in the three loci (rs2231142 in ABCG2, rs16890979 in SCL2A9, and rs1165205 in SLC17A3) are taken forward to the FBAT analyses, where we can use 0.05/3 as the significance level, all the three top SNPs in SCL2A9, ABCG2 and SCL17A3 reach the significance level. The results of the three top SNPs are presented in Table 7.

**Table 7:** Top SNP in each gene identified from the three-stage analyses of Uric acid levels in FHS SHARe project.

SNP	Chr	Position	Gene	MAF	LM pval	LME pval	FBAT pval	Direction	Three-stage pval	Three-stage pval for top SNPs
rs1165205	6	25978521	SLC17A3	0.46	3.2E-11	5.6E-10	7.1E-03	---	1.0E+00	2.1E-02

rs2231142	4	89271347	ABCG2	0.11	2.4E-23	9.0E-20	5.6E-11	+++	8.3E-09	1.7E-10

rs16890979	4	9531265	SLC2A9	0.23	3.4E-88	1.6E-76	8.3E-23	---	1.2E-20	2.5E-22

1) Direction: the three signs are the direction of changes in multivariable adjusted uric acid per minor allele in the LM, LME and FBAT analyses, respectively.

2) Three-stage pval: Three-stage p-values adjusting for the multiple testing of all SNPs with p-value < 5 × 10^-8 ^in the second stage.

3) Three-stage pval for top SNPs: Three-stage p-values adjusting for the multiple testing of only the top SNP in each gene among all SNPs with p-value < 5 × 10^-8 ^in the second stage.

## Discussion

We have proposed a three-stage strategy to conduct GWA analyses on quantitative traits for family data. This strategy consists of LM as the first stage, LME accounting for familial relatedness but without correction for admixture as the second stage, and FBAT as the final stage. Simulation studies have shown that this approach is more powerful than single stage FBAT and is robust to population admixture.

When there is no population admixture, our simulation results show that FBAT, LME and the two-stage approach had correct type I error rates even for non-Normal phenotypes and for SNPs with MAF as low as 0.005. This would justify the use of two-stage approach by applying LME to the SNPs screened by LM and the benefit is the time-saving efficiency almost without power loss in GWA studies except power loss may occur to non-normal phenotypes [15]. In our simulation studies, it took 283 seconds to complete LME analyses but only 2 seconds to complete LM analyses of 100 SNPs on a single Linux processor (2 × Dual-Core AMD Opteron(tm) Processor 2218 HE and total 12 GB RAM). So a genome-wide association analyses of 2.5 million Hapmap SNPs will require 1965 hours (82 days) if using LME, but only 210 hours (9 days) on such a single processor if using the two-stage approach since about 10% SNPs are analyzed using LME in the two stage approach. When the population admixture exists, our simulation results show that the three-stage approach inherits FBAT's robustness to population admixture and LME's good power to detect the associated SNPs. We also found that using PC of all the SNPs in a GWA study may not always be able to adjust for the population admixture that involves only a subset of SNPs. Although it is not clear what pattern the admixture is most likely to follow in a given population, it is conceivable that there could be difference between subpopulations in limited chromosome regions. When the admixture effect only involves a subset of SNPs, our approach is more robust to population admixture than LME adjusting for PC estimated using all SNPs and is more powerful than LME adjusting for PC estimated using admixture SNPs.

For genome-wide scan with dense SNPs, many significant SNPs in LME analyses may be associated with a single locus. When all of these SNPs are included in FBAT analyses, Bonferroni correction for FBAT is apparently too conservative. One modification of our approach is to identify SNPs independently associated with the phenotype using stepwise selection or other selection scheme among the significant SNPs detected by LME. Then only conduct FBAT analyses on selected SNPs independently associated with the phenotype in the LME analyses. By doing this, Bonferroni correction for FBAT would be much less conservative. In our application to uric acid level GWA analysis, the modified three-stage approach confirmed the three genome-wide significantly associated loci (SCL2A9, ABCG2 and SCL17A3) identified by LME in FHS SHARe project.

A potential modification of the three-stage approach to avoid power loss in FBAT is to include one more stage by applying quantitative trait linkage disequilibrium (QTLD [16]) to test stratification/admixture for the SNPs identified by the second stage LME. If there is no admixture, the tested SNP may be claimed as a signal. Otherwise, FBAT is then applied to test the SNPs with admixture for association.

FBAT is known for the robustness against admixture and the lack of power compared to population-based methods. To circumvent multiple testing and improve its power in GWA analyses, two-stage FBAT approaches were proposed [7,17]. In brief, the first screening stage regresses the offspring phenotype on the offspring's expected genotype, given parental genotypes; the second stage in [17] then applies FBAT to the best 10 SNPs from the screening stage and the correction for multiple testing is applied only to the second stage, while the second stage in [7] ranks all SNPs based on the results from the first stage and tests the *i*-th ranked SNP with the significance level *w*_*i*_*α*, where Σ*w*_*i *_= 1, *w*_1 _> 0 and *α *is the genome-wide significance level. [18] presents an application of the two-stage FBAT approach of [17]. The two-stage FBAT approach and our three-stage approach share the similarities of 1) the initial stages are not robust to admixture; 2) FBAT is the last stage. However, the parental phenotype data are not used in the two-stage FBAT approach which may cause loss in power.

Even though our main interest focuses on quantitative traits, similar strategies can be applied to dichotomous traits, where the first stage uses logistic regression, the second stage uses generalized estimating equations (GEE) [19], and the third stage uses FBAT. In the second stage, the GEE approach can be applied with an independence working correlation matrix or an exchangeable (compound symmetry) working correlation matrix and empirical sandwich estimators, since GEE is known to be robust against misspecification of the correlation structure models.

## Conclusions

For GWA analyses on quantitative traits with family data, our three-stage approach provides another appealing method robust to population admixture, in addition to using LME adjusting for PC.

## Authors' contributions

All authors contributed to the design of the overall strategy. MHC conducted all the analyses. QY and MHC drafted the manuscript. All authors read and approved the final manuscript.

## References

[B1] Price AL Patterson NJ Plenge RM Weinblatt ME Shadick NA Reich D Principal components analysis corrects for stratification in genome-wide association studies Nat Genet 2006 38 904 909 10.1038/ng1847 16862161

[B2] Kimmel G Jordan MI Halperin E Shamir R Karp RM A randomization test for controlling population stratification in whole-genome association studies Am J Hum Genet 2007 81 5 895 905 10.1086/521372 17924333 PMC2265648

[B3] Spielman RS Mcginnis RE Ewens WJ Transmission Test for Linkage Disequilibrium - the Insulin Gene Region and Insulin-Dependent Diabetes-Mellitus (Iddm) Am J Hum Genet 1993 52 3 506 516 8447318 PMC1682161

[B4] Rabinowitz D Laird N A unified approach to adjusting association tests for population admixture with arbitrary pedigree structure and arbitrary missing marker information Hum Hered 2000 50 4 211 223 10.1159/000022918 10782012

[B5] Abecasis GR Cardon LR Cookson WOC Sham PC Cherny SS Association analysis in a variance components framework Genet Epidemiol 2001 21 S341 346 11793695 10.1002/gepi.2001.21.s1.s341

[B6] Amos CI de Andrade M Genetic linkage methods for quantitative traits Stat Methods Med Res 2001 10 1 3 25 10.1191/096228001677031143 11329691

[B7] Ionita-Laza I McQueen MB Laird NM Lange C Genomewide weighted hypothesis testing in family-based association studies, with an application to a 100K scan Am J Hum Genet 2007 81 3 607 614 10.1086/519748 17701906 PMC1950836

[B8] Almasy L Blangero J Multipoint quantitative-trait linkage analysis in general pedigrees Am J Hum Genet 1998 62 5 1198 1211 10.1086/301844 9545414 PMC1377101

[B9] genetics Population Genetics http://cran.r-project.org/web/packages/genetics/

[B10] Balding DJ Nichols RA A method for quantifying differentiation between populations at multi-allelic loci and its implications for investigating identity and paternity Genetica 1995 96 3 12 10.1007/BF01441146 7607457

[B11] Balding DJ Likelihood-based inference for genetic correlation coefficients Theor Popul Biol 2003 63 3 221 230 10.1016/S0040-5809(03)00007-8 12689793

[B12] Chen MH Yang Q GWAF: an R package for genome-wide association analyses with family data Bioinformatics 2010 26 4 580 581 http://cran.r-project.org/web/packages/GWAF/10.1093/bioinformatics/btp710 20040588 PMC2852219

[B13] kinship: mixed-effects Cox models, sparse matrices, and modeling data from large pedigrees http://cran.r-project.org/web/packages/kinship/

[B14] Dehghan A Köttgen A Yang Q Hwang SJ Kao WL Rivadeneira F Boerwinkle E Levy D Hofman A Astor BC Benjamin EJ van Duijn CM Witteman JC Coresh J Fox CS Association of three genetic loci with uric acid concentration and risk of gout: a genome-wide association study Lancet 2008 372 1953 61 10.1016/S0140-6736(08)61343-4 18834626 PMC2803340

[B15] Lange C DeMeo DL Laird NM Power and design considerations for a general class of family-based association tests: quantitative traits Am J Hum Genet 2002 71 1330 1341 10.1086/344696 12454799 PMC378574

[B16] Havill LM Dyer TD Richardson DK Mahaney MC Blangero J The quantitative trait linkage disequilibrium test: a more powerful alternative to the quantitative transmission disequilibrium test for use in the absence of population stratification BMC genet 2005 6 Suppl 1 S91 10.1186/1471-2156-6-S1-S91 16451707 PMC1866688

[B17] Lange C DeMeo D Silverman EK Weiss ST Laird NM Using the noninformative families in family-based association tests: a powerful new testing strategy Am J Hum Genet 2003 73 801 811 10.1086/378591 14502464 PMC1180603

[B18] Herbert A Gerry NP McQueen MB Heid IM Pfeufer A Illig T Wichmann HE Meitinger T Hunter D Hu FB Colditz G Hinney A Hebebrand J Koberwitz K Zhu X Cooper R Ardlie K Lyon H Hirschhorn JN Laird NM Lenburg ME Lange C Christman MF A common genetic variant is associated with adult and childhood obesity Science 2006 312 279 283 10.1126/science.1124779 16614226

[B19] Liang KY Zeger SL Longitudinal data analysis using generalized linear models Biometrika 1986 73 13 22 10.1093/biomet/73.1.13

